# Non-cancer-related Deaths in Cancer Survivors: A Nationwide Population-based Study in Japan

**DOI:** 10.2188/jea.JE20240230

**Published:** 2025-03-05

**Authors:** Yasufumi Gon, Ling Zha, Toshitaka Morishima, Yasuyoshi Kimura, Kanako Asai, Haruka Kudo, Tsutomu Sasaki, Hideki Mochizuki, Isao Miyashiro, Tomotaka Sobue

**Affiliations:** 1 Department of Neurology, Osaka University Graduate School of Medicine, Osaka, Japan; 2 Academic Clinical Research Center, Osaka University Hospital, Osaka, Japan; 3 Cancer Control Center, Osaka International Cancer Institute, Osaka, Japan; 4 Department of Social Medicine, Environmental Medicine and Population Science, Osaka University Graduate School of Medicine, Osaka, Japan; 5 StemRIM Institute of Regeneration-Inducing Medicine, Osaka University Graduate School of Medicine, Osaka, Japan

**Keywords:** cancer, survivor, non-cancer-related mortality, cohort study

## Abstract

**Background:**

Advancements in cancer care have improved survivorship, potentially leading to changes in mortality causes. This study aimed to investigate the causes of death among cancer survivors, specially focusing on non-cancer-related mortality.

**Methods:**

This nationwide population-based cohort study analyzed the causes of death based on the time since cancer diagnosis using data from the National Cancer Registry in Japan between January 2016 and December 2019. Non-cancer-related deaths were identified, and mortality risks associated with non-cancer diseases were compared to those of the Japanese general population using standardized mortality ratios (SMRs) with 95% confidence intervals (CIs). The follow-up period was up to 4 years after cancer diagnosis.

**Results:**

A total of 3,990,661 patients (45.8% women) were included in the analysis, yielding 6,237,269 person-years of follow-up. Of these, 1,001,857 (25.1%) patients died during the study period. Cancer-related and non-cancer-related causes accounted for 86.6% and 13.4% of deaths, respectively. The proportion of non-cancer-related deaths increased from 10.2% at 6 months to 31.6% at 4 years after cancer diagnosis. Heart disease (21.8%), cerebrovascular disease (9.8%), and pneumonia (9.1%) were the leading cause of non-cancer-related deaths: the SMRs for these diseases were 2.69 (95% CI, 2.66–2.72), 2.07 (95% CI, 2.03–2.10), and 2.41 (95% CI, 2.36–2.45), respectively. The SMR for suicide was 1.81 (95% CI, 1.74–1.89); however, it lost significance in males and females 2 and 2.5 years after cancer diagnosis, respectively.

**Conclusion:**

The proportion of non-cancer-related deaths among cancer patients has increased over time, emphasizing the need to manage cancer and its comorbidities carefully.

## INTRODUCTION

Advancements in cancer care have improved patient survival,^1^ leading to more diverse causes of death.^2^ Cancer remains the primary cause of death, but non-cancer diseases also contribute to mortality.^2^^–^^4^ Cardiovascular diseases are the leading causes of non-cancer-related death among cancer patients, with higher mortality risk than the general population.^2^^,^^5^^–^^11^ Other conditions, such as infectious diseases and neurodegenerative disorders, show similar trends.^2^^,^^12^^,^^13^ With increasing comorbidities in aging cancer survivors, clinicians are expected to manage cancer and comorbidities.^14^

The cause of death varies considerably depending on the age at cancer diagnosis.^2^ Data from aging populations will aid in global cancer control as the world’s population continues aging.^15^ Japan has one of the most rapidly aging populations in the world. The number of elderly individuals (aged ≥65 years) in Japan is projected to continue rising, reaching 28.7% in 2025 and 35.7% in 2050.^16^ The current scenario in Japan can provide insights into scenarios that the rest of the world may encounter in the future. We have conducted the Neoplasms and Other Causes of Death (NANDE) study to investigate the causes of death among cancer survivors in Japan.^5^^,^^10^^,^^13^^,^^17^^–^^21^ However, the NANDE study was restricted to certain areas of Japan. The National Cancer Registry (NCR) of Japan was not available for research purposes at the time of conducting the NANDE study.

This study aimed to investigate the causes of death among cancer patients in Japan using the NCR data, with a focus on non-cancer-related death in cancer patients.

## METHODS

### Ethics approval and consent to participate

The Institutional Review Board of Osaka University Hospital approved this study (approval number: 21438) and waived the requirement for informed consent due to the use of anonymized data.

### Study design and participants

This nationwide population-based cohort study was conducted as an extension of the NANDE study.^5^^,^^10^^,^^13^^,^^17^^–^^21^ Data of cancer patients registered in the NCR, covering the entire population of Japan, were used. The NCR was launched in 2016 based on the Act on the Promotion of Cancer Registries.^22^ The survival information of the patients registered in the NCR, including the cause of death, is regularly updated. Deceased patients have their cause of death recorded. The data from patients diagnosed with cancer between January 1, 2016, and December 31, 2019, was accessed with the permission of the NCR Information Provision and Review Committee of the National Cancer Center. The exclusion criteria were as follows: (1) uncertain sex, (2) uncertain age at cancer diagnosis, (3) death certificate notification or death certification only, and (4) registration for second and subsequent cancers (eFigure 1).

### Variable definition

The age at diagnosis was grouped into six categories: ≤39, 40–49, 50–59, 60–69, 70–79, and ≥80 years. The stage at diagnosis was classified into seven categories: (i) intraepithelial (abnormal cells were present but have not spread to nearby tissues), (ii) localized (cancer was limited to the organ where it originated, with no sign of spread), (iii) lymph node metastasis (cancer had spread to regional lymph nodes), (iv) infiltration to adjacent organs (cancer had spread to nearby tissues or organs), (v) distant metastasis (cancer had metastasized to distant parts of the body), (vi) unstaged/unknown (there is insufficient information to determine the stage), and (vii) not applicable (missing data on the stage).^23^ The International Classification of Diseases for Oncology, Third Edition, was used to code the cancer. The International Classification of Diseases, Tenth Edition, was used to register the causes of death in the NCR based on death certificates. The causes of death, including heart disease, senility, cerebrovascular disease, pneumonia, aspiration pneumonia, accidents (external caused injuries), renal failure, and Alzheimer’s disease, were classified according to the Japanese mortality statistics.^24^ Furthermore, diseases with high frequencies were investigated as causes of non-cancer-related deaths in the study cohort. eTable 1 and eTable 2 detail the assignment of codes.

### Statistical analysis

The causes of death were analyzed according to the time since cancer diagnosis. Survival of <1 month was recorded as 0 days in the NCR. Therefore, patients with 0 survival months in the NCR were assigned a value of 0.5 months.^25^^,^^26^ The observation period spanned from January 1, 2016, to December 31, 2019. The follow-up period started on the date of cancer diagnosis and ended on the date of death or December 31, 2019, whichever came first.

To examine the causes of death among cancer patients, we classified them into three categories: (i) index cancer-related death, comprising patients who died from the originally diagnosed cancer; (ii) non-index cancer-related death, comprising patients who died from cancers other than the originally diagnosed cancer; and (iii) non-cancer-related death, comprising patients who died from any medical cause not coded as cancer. The breakdown of non-cancer-related deaths was also investigated.

To compare the risk of non-cancer-related death among cancer patients to that in the Japanese general population, the standardized mortality ratios (SMRs) and their 95% confidence intervals (CIs) were calculated as the ratio of the observed to the expected number of deaths. The observed number of deaths was obtained from the database. The expected number was calculated by summing the products of multiplying the population in each 5-year age group of the study cohort by the national cause-specific mortality rate for the corresponding sex, age group, and calendar year in Japan. Information concerning both the national population and the number of deaths, including in patients with and without cancer, is available at the Portal Site of Official Statistics of Japan (https://www.e-stat.go.jp/en).

All statistical analyses were performed using the Stata 17/MP software (StataCorp, College Station, TX, USA). All tests were two sided, and *P* < 0.05 was considered statistically significant.

## RESULTS

### Patient characteristics

Among the 4,686,949 patients registered in the NCR during the study period, 3,990,661 patients (45.8% women) were included in the analysis, yielding 6,237,269 person-years of follow-up. Table 1 presents the demographic characteristics.

**Table 1.** Demographic characteristics of the study cohort

	*N*	%
Sex		
Female	1,826,575	45.8%
Male	2,164,086	54.2%
Age at diagnosis, years		
≤39	158,233	4.0%
40–49	272,400	6.8%
50–59	414,393	10.4%
60–69	928,879	23.3%
70–79	1,230,320	30.8%
≥80	986,436	24.7%
Period of diagnosis		
2016	1,003,894	25.2%
2017	998,756	25.0%
2018	991,993	24.9%
2019	996,018	25.0%
Stage at diagnosis		
Intraepithelial	399,046	10.0%
Localized	1,671,185	41.9%
Lymph node metastasis	327,146	8.2%
Infiltration to adjacent organs	471,678	11.8%
Distant metastasis	642,145	16.1%
Unstaged/Unknown	122,809	3.1%
Not applicable	356,652	8.9%
Cancer type		
Colorectal	688,957	17.3%
Stomach	461,411	11.6%
Lung	418,823	10.5%
Breast	405,162	10.2%
Prostate	328,821	8.2%
Uterus	197,130	4.9%
Bladder	150,118	3.8%
Pancreas	144,613	3.6%
Liver	130,772	3.3%
Lymphoma	123,810	3.1%
Hematologic (excluding lymphoma)	122,924	3.1%
Skin	110,685	2.8%
Renal	106,136	2.7%
Brain	91,772	2.3%
Esophagus	90,845	2.3%
Lip, oral cavity, and pharynx	78,060	2.0%
Gallbladder	76,534	1.9%
Thyroid	65,709	1.6%
Ovary	47,684	1.2%
Larynx	18,729	0.5%
Bone	16,229	0.4%
Others	115,837	2.9%

### Cause of death according to the number of years after cancer diagnosis

**Figure 1.**
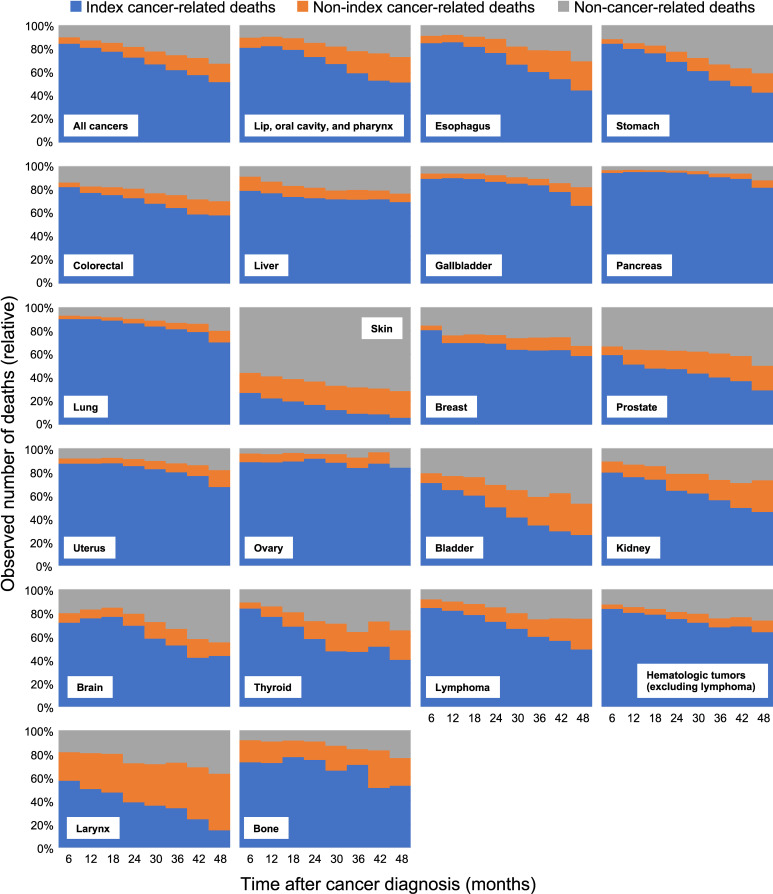
Proportion of deaths according to the time since cancer diagnosis and cancer types. The vertical axis indicates the observed number of deaths (relative) who died at the time of observation, and the horizontal one shows the time since cancer diagnosis in months. For cancers with high survival rates, such as skin, prostate, and breast cancers, the proportion of non-cancer-related deaths was higher than that for other cancers. Conversely, for cancers with low survival rates, including pancreatic, lung, and liver cancers, cancer-related deaths accounted for the majority of deaths.

Among 1,001,857 patients who died during the study period, 801,763 (80%), 65,948 (6.6%), and 134,146 (13.4%) died of index cancer, non-index cancer, and non-cancer causes, respectively. Figure 1 shows the proportion of deaths post-diagnosis. Regarding all cancers, cancer-related deaths, including index and non-index cancer-related deaths, accounted for approximately 90% of all deaths immediately after cancer diagnosis. The proportion decreased to 68.4% at 4 years. In contrast, the prevalence of non-cancer-related deaths increased from 10.2% at 6 months to 31.6% at 4 years after cancer diagnosis. When analyzed by cancer type, index cancer-related deaths were more common among cancer patients with low survival rates, such as pancreatic and lung cancers. In contrast, non-cancer-related deaths were more prevalent among cancer patients with high survival rates, such as skin and prostate cancers.

### Comparison between the risk of non-cancer-related death in cancer patients and the general population

**Figure 2.**
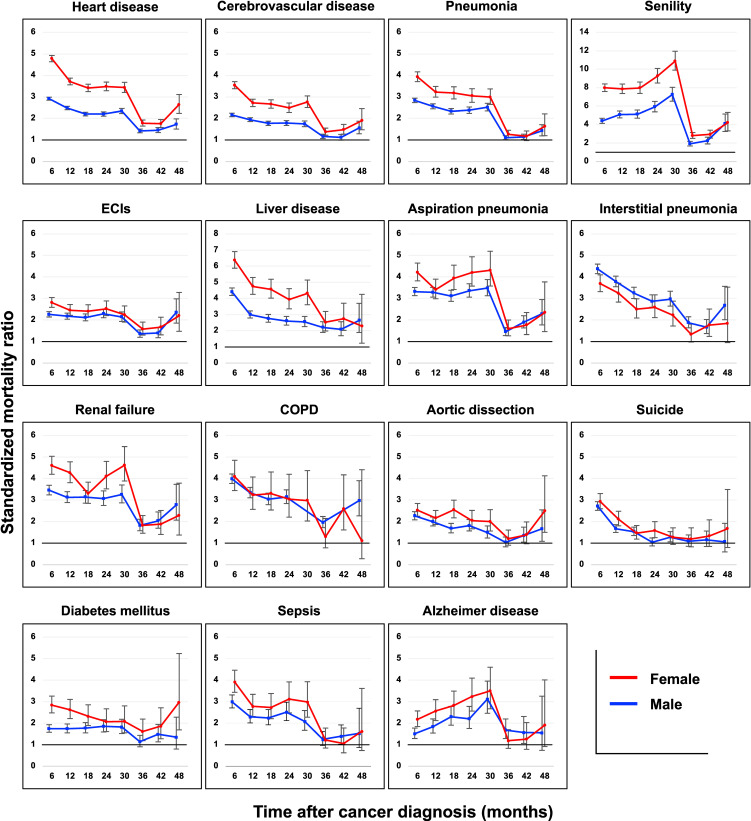
Standardized mortality ratios for the leading causes of non-cancer-related deaths. The panels show standardized mortality ratios with 95% confidence intervals calculated according to the time post-diagnosis and analyzed by the major causative diseases for non-cancer-related deaths. The blue and red lines represent males and females, respectively. The error bars in the panels represent 95% confidence intervals. COPD, chronic obstructive pulmonary disease; ECIs, externally caused injuries.

Table 2 lists the leading causes of non-cancer-related death, mortality rates, and SMRs. Heart disease was the leading cause of non-cancer-related death (21.8%), followed by cerebrovascular disease (9.8%), and pneumonia (9.1%). Suicide was the 12th cause of non-cancer-related death. Figure 2 illustrates the SMRs for the leading causes of non-cancer-related death. In many diseases, the SMRs were higher or equivalent in females compared to males. However, in interstitial pneumonia, the SMR was higher in males than in females. The SMR for suicide increased immediately after cancer diagnosis; however, the SMR decreased over time, becoming statistically non-significant after 2 and 2.5 years for males and females, respectively.

**Table 2.** Non-cancer-related deaths among the patients with cancer

	*N*	%	Mortality rate^a^ (95% CI)	SMR (95% CI)
Heart disease	29,273	21.8%	469.31 (463.96–474.72)	2.69 (2.66–2.72)
Cerebrovascular disease	13,123	9.8%	210.38 (206.81–214.01)	2.07 (2.03–2.10)
Pneumonia	12,208	9.1%	195.68 (192.24–199.18)	2.41 (2.36–2.45)
Senility	7,961	5.9%	127.64 (124.86–130.47)	5.49 (5.37–5.62)
Externally caused injuries	5,993	4.5%	96.07 (93.67–98.53)	2.18 (2.12–2.23)
Liver disease	5,078	3.8%	81.09 (79.14–83.62)	3.53 (3.44–3.63)
Aspiration pneumonia	4,977	3.7%	79.78 (77.59–82.03)	3.05 (2.97–3.14)
Interstitial pneumonia	4,932	3.7%	79.04 (76.87–81.28)	3.28 (3.19–3.37)
Renal failure	4,202	3.1%	67.37 (65.36–69.44)	3.23 (3.13–3.33)
Chronic pulmonary obstructive disease	3,866	2.9%	61.97 (60.04–63.95)	3.21 (3.11–3.31)
Aortic dissection or aneurysm	2,426	1.8%	38.90 (37.38–40.47)	1.94 (1.86–2.02)
Suicide	2,275	1.7%	36.47 (35.01–38.00)	1.81 (1.74–1.89)
Diabetes mellitus	1,891	1.4%	30.32 (28.98–31.72)	1.88 (1.80–1.97)
Sepsis	1,670	1.2%	26.76 (25.01–28.07)	2.48 (2.37–2.60)
Alzheimer’s disease	1,118	0.8%	17.92 (16.90–19.01)	2.09 (1.97–2.21)
Others	33,153	24.7%	—	—

CI, confidence interval; SMR, standardized mortality ratio.

^a^Per 100,000 person-years.

## DISCUSSION

The finding that non-cancer-related deaths in cancer patients increased with the time since cancer diagnosis was consistent with previous studies.^2^^–^^5^ Advancements in cancer care and the aging population have led to an increased aging demographic among cancer patients, who often presenting with comorbidities beyond cancer. Our study emphasizes the importance of the careful management of non-cancerous conditions in cancer patients.

In a previous study using the NCR in Japan, a decrease in the SMR for cardiovascular disease was observed over the years, reaching a level almost equivalent to that of the general population by the second year.^24^ In the present study, mortality risks for heart and cerebrovascular diseases remained consistently high. We propose three explanations for this difference. First, we separately defined cardiovascular disease, distinguishing between heart (I00–I09, I11, I13, and I20–I52) and cerebrovascular diseases (I60–I69), excluding other cardiovascular diseases. In contrast, the previous study included all cardiovascular codes (I00–I99).^25^ Second, we included all cancer types in calculating the SMR, whereas the previous research excluded brain tumors from the analysis of cardiovascular mortality,^24^ which have a high SMR for cardiovascular disease.^5^^–^^7^^,^^10^ Third, the observation period of the present study was longer than that of the previous study.^25^ The proportion of non-cancer-related deaths increased with the time after cancer diagnosis, resulting in a higher SMR for heart and cerebrovascular diseases. Previous studies have suggested that the increased risk of cardiovascular disease mortality in cancer patients persists over the long term.^2^^,^^5^^,^^7^^,^^8^^,^^10^ Thus, cardiovascular diseases remain a significant concern for cancer survivors and warrant continued attention.

The SMRs for heart and cerebrovascular diseases sharply declined at 30–36 months, then subsequently increased. Heart and cerebrovascular diseases showed a nadir at 30–36 months in the NANDE study, followed by an increase.^5^^,^^10^ It appears that the mortality risk is lowest around 3 years after the cancer diagnosis, then rises thereafter. A sharp decline in the SMRs for Alzheimer’s disease and senility was also observed at 30–36 months. Among patients who survived more than 2.5 years, there were many younger individuals. Therefore, it is possible that diseases, such as Alzheimer’s disease and senility, were less selected as causes of death, resulting in lower SMR. However, this explanation is insufficient to fully account for the observed results. Further investigation is needed to understand these findings.

The mortality risk associated with suicide among cancer patients was higher than that in the general population. This aligns with the findings of previous studies from Japan^25^^,^^26^ and other countries.^27^^–^^32^ A prior Japanese study had a 2-year observation period,^25^^,^^26^ while the present study extended it up to 4 years, enabling a longer evaluation of suicide risk post-cancer diagnosis. A statistically significance decrease in suicide risk was observed after 2 years in males and 2.5 years in females, suggesting that although suicide risk is heightened shortly after cancer diagnosis, it tends to decrease over time. This trend is consistent with findings from a Surveillance, Epidemiology, and End Results database study.^32^ Suicide risk among cancer patients varies by time since diagnosis and sex, necessitating individualized care for each patient.

Another distinctive finding was an increased risk of death due to senility. Senility was the fourth leading cause of non-cancer death, with a mortality risk approximately 5.5 times higher than that in the general population. This result was higher than we expected. Senility is recorded as the in Japan only when there is no other apparent cause of death in elderly individuals.^33^ However, these instructions may not be followed accurately in all cases. Only senility is listed on the death certificate in some cases, whereas senility is listed on the death certificate in other cases as the family considers it a peaceful death and wishes it to be so.^33^ An increased number of deaths is being attributed to senility in facilities housing older adults.^33^ Thus, it is possible that senility is recorded as the cause of death on the death certificate despite the presence of cancer.

A decrease in the SMR for infectious diseases, such as pneumonia and sepsis, was observed as the duration since cancer diagnosis. One of the possible explanations of this finding is complications arising from cancer treatment. For instance, neutropenia, the most severe hematological toxicity of chemotherapy, increases the risk of infections.^34^ The risk of local infections is also high during the post-cancer surgery period.^35^^,^^36^

Our study has several limitations. First, the cause of death in the NCR was recorded based on the death certificates, which may be inaccurate at times.^37^ Nonetheless, this method is currently the most reliable information source of cause of death and widely used.^2^^,^^3^^,^^5^^–^^10^^,^^12^^,^^13^^,^^17^^–^^21^^,^^25^^–^^29^^,^^31^^,^^32^^,^^36^ Thus, our study provides key findings. Second, the biases associated with the application of SMR may exist, particularly in relation to rare events, heterogeneous populations, and unmeasured confounders. Finally, the observation period for this study extended until December 2019, representing the longest duration available in the datasets accessed; however, the maximum follow-up period for patients diagnosed in 2019 was limited to 1 year. Our findings underscore the impact up to 1 year after cancer diagnosis.

In conclusion, cancer survivors are at high risk of non-cancer-related deaths. Cardiovascular disease, including heart and cerebrovascular diseases, accounted for one-third of non-cancer-related deaths among cancer survivors. The mortality risk associated with suicide was high immediately after cancer diagnosis but was equivalent to that of the general population after 2–2.5 years later. Recognizing and addressing these risks in cancer care will improve patient outcomes.

## SUPPLEMENTARY MATERIAL

eTable 1. The assignment of codes for primary tumor site

eTable 2. The assignment of codes for death

eFigure 1. Flow chart of eligible patients
