# Supplementary material for: Non-cancer-related Deaths in Cancer Survivors: A Nationwide Population-based Study in Japan
Source: J Epidemiol. 2025 Mar 5;35(3):147–53. doi: 10.2188/jea.JE20240230 (PMC11821378; doi:10.2188/jea.JE20240230)

eTable 1. The assignment of codes for primary tumor site

		Primary cancer site	
		ICD-O-3[a] morphology	ICD-O-3[a]
All			
Lip, oral cavity, and pharynx			C00-C14
Esophagus			C15
Stomach			C16
Colorectal			C18-C20
Liver and intrahepatic bile duct			C22
Gallbladder and other biliary tract			C23, C24
Pancreas			C25
Larynx			C32
Trachea, bronchus, and lung			C33, C34
Bone and soft tissue			C40, C41, C47, C49
Skin			C43, C44
Breast			C50
Uterus			C53-C55
Ovary			C56
Prostate			C61
Bladder			C67
Renal and urinary tract			C64-C66, C68
Brain			C700, C71, C751-753
Thyroid			C73
Lymphoma	Non-Hodgkin lymphoma	959-964, 967-972, 974-975	
	Hodgkin lymphoma	965-966	
Hematologic tumors (excluding lymphoma)		973, 976, 980-998	
Other malignant neoplasms			Others

[a] International Classification of Diseases for Oncology, Third edition

eTable 2. The assignment of codes for death

		ICD-10[a]
Cancer		
Lip, oral cavity, and pharynx		C00-C14
Esophagus		C15, D001
Stomach		C16
Colorectal		C18-C20, D010-D012
Liver and intrahepatic bile duct		C22, D015[b]
Gallbladder and other biliary tract		C23, C24, D015[b]
Pancreas		C25
Larynx		C32
Trachea, bronchus, and lung		C33, C34, D021, D022
Bone and soft tissue		C40, C41, C47, C49
Skin		C43, C44, D030-D049
Breast		C50, D05
Uterus		C53-C55, D06
Ovary		C56
Prostate		C61, D075
Bladder		C67, D090
Renal and urinary tract		C64-C66, C68, D41
Brain		C700, C71, C751-C753, D320-D326, D327-D339, D420, D429-D433, D437, D439
Thyroid		C73, D440
Lymphoma	Non-Hodgkin lymphoma	C82-C85
	Hodgkin lymphoma	C81
Hematologic tumors (excluding lymphoma)		C86-C96, D45-D47
Other malignant neoplasms		Others
Noncancer disease		
Heart disease		I00-I09, I11, I13, I20-52
Cerebrovascular disease		I60-I69
Pneumonia		J12-J18
Senility		R54
Externally caused injuries		V01-X59, Y10-Y34
Liver Disease		K70-K76
Aspiration pneumonia		J69
Interstitial pneumonia		J84
Renal failure		N17-N19
Chronic pulmonary obstructive disease		J41-J43
Aortic dissection or aneurysm		I71
Suicide		X60-X84, Y87
Diabetes mellitus		E10-E14
Sepsis		A40, A41
Alzheimer's disease		G30
Other diseases		A00-U99, not in the above

[a] International Classification of Diseases, Tenth revision

[b] Cases in which the ICD-10 for cause of death was registered as D015 were assigned according to the location of the primary cancer site.

eFigure 1. Flow chart of eligible patients

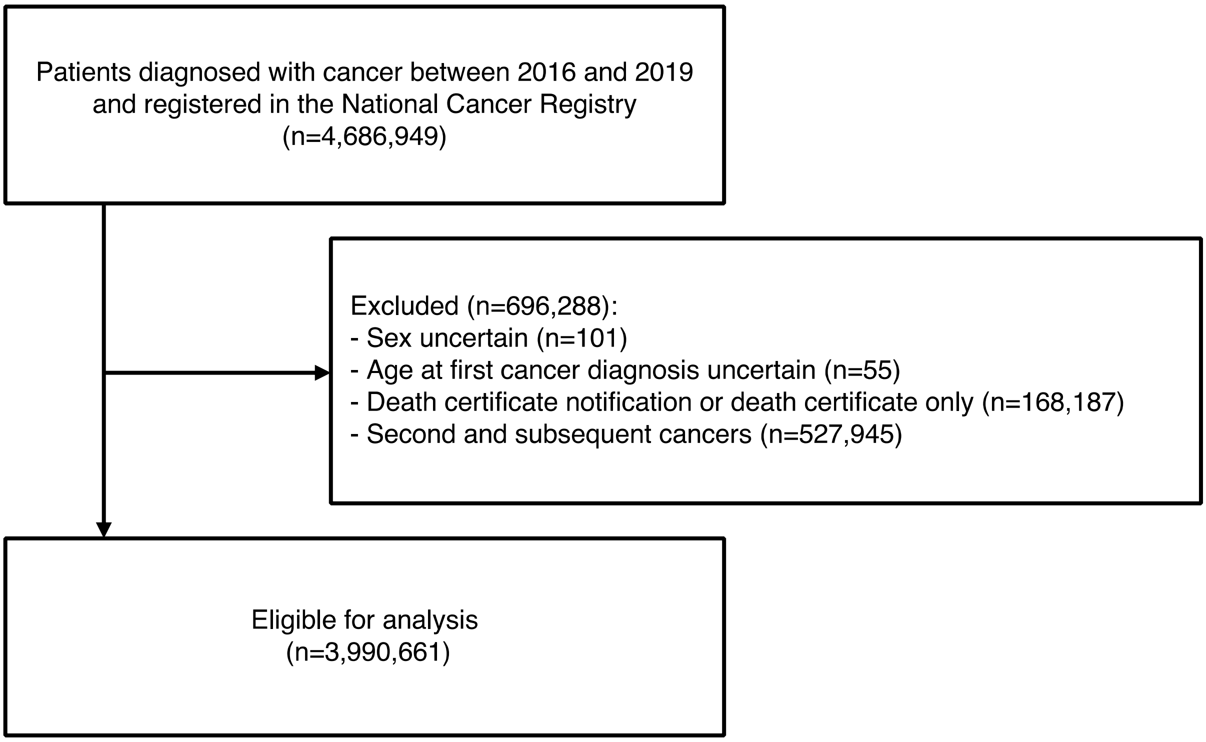

Supplement: Supplementary file 1 [file je-35-147-s001.pdf]
